# Association of Intramyocardial Hemorrhage With Inflammatory Biomarkers in Patients With ST-Segment Elevation Myocardial Infarction

**DOI:** 10.1016/j.jacadv.2025.101647

**Published:** 2025-03-12

**Authors:** Christina Tiller, Martin Reindl, Magdalena Holzknecht, Ivan Lechner, Fritz Oberhollenzer, Sebastian von der Emde, Alex Kaser, Agnes Mayr, Mathias Pamminger, Can Gollmann-Tepeköylü, Axel Bauer, Bernhard Metzler, Sebastian J. Reinstadler

**Affiliations:** a Cardiology and Angiology, Medical University of Innsbruck, University Clinic of Internal Medicine III, Innsbruck, Austria; b Medical University of Innsbruck, University Clinic of Radiology, Innsbruck, Austria; c University Clinic of Cardiac Surgery, Medical University of Innsbruck, Innsbruck, Austria

**Keywords:** cardiac magnetic resonance, inflammation, intramyocardial hemorrhage, risk stratification, ST-segment elevation myocardial infarction

## Abstract

**Background:**

Ischemia-reperfusion (I/R) injury patterns detected by cardiac magnetic resonance imaging after percutaneous coronary intervention (PCI) have important prognostic implications and trigger inflammatory processes that can further enhance myocardial tissue damage.

**Objectives:**

The authors aimed to investigate the association of circulating inflammatory markers and I/R injury patterns in patients with ST-segment elevation myocardial infarction (STEMI).

**Methods:**

This observational study included 456 STEMI patients. Peripheral venous blood samples were drawn 48 hours after PCI for analysis of high-sensitivity C-reactive protein (hs-CRP), white blood cell count (WBCc), and interleukin (IL)-6. The presence of I/R injury was defined by the detection of intramyocardial hemorrhage (IMH) according to cardiac magnetic resonance T2∗. Clinical endpoint was the occurrence of major adverse cardiac events, defined as composite of all-cause death, nonfatal reinfarction, and new congestive heart failure.

**Results:**

IMH was present in 150 (33%) patients. Hs-CRP (OR: 2.89; 95% CI: 1.96-4.26; *P* < 0.001), WBCc (OR: 1.32; 95% CI: 1.04-1.67; *P* = 0.021), and IL-6 (OR: 1.86; 95% CI: 1.38-2.51; *P* < 0.001) were associated with presence of IMH. Only hs-CRP was independently associated with IMH (OR: 1.95; 95% CI: 1.30-2.93; *P* = 0.001) after adjustment for other clinical parameters. Furthermore, patients with hs-CRP levels above the median (>26.4 mg/L) were more likely to experience major adverse cardiac events (12% vs 4%, *P* = 0.002) during a median follow-up of 12 (Q1-Q3: 12-13) months.

**Conclusions:**

In patients with STEMI treated with primary PCI, inflammatory parameters including hs-CRP, WBCc, and IL-6 were significantly associated with I/R injury as defined by IMH. After adjustment for other factors, hs-CRP was the only independent inflammatory biomarker associated with IMH.

Although early reperfusion by percutaneous coronary intervention (PCI) is the treatment of choice in acute ST-segment elevation myocardial infarction (STEMI), it causes additional myocardial damage through a series of pathological processes including inflammation and microvascular injury.^1^ This phenomenon is referred to as ischemia-reperfusion (I/R) injury.^2^ In the array of pathophysiological mechanisms contributing to I/R injury, inflammation appears to play a crucial role in the final extent of myocardial damage after STEMI.^3^ Myocardial injury triggers a significant release of inflammatory markers which are mandatory for infarct healing but may also contribute to further damage to the ischemic myocardium.^4^ High-sensitivity C-reactive protein (hs-CRP), interleukin (IL)-6, and white blood cell count (WBCc) are key clinical inflammatory markers in this setting and have been studied well.^5^, ^6^, ^7^, ^8^ Particularly elevated CRP levels are related to a greater extent of both myocardial and microvascular injury and subsequent clinical outcome.^9^^,^^10^ Experimental data suggest that CRP is not only associated with infarct injury but may also play a causal role in the propagation of this myocardial tissue damage.^11^, ^12^, ^13^ However, the exact role of inflammation, especially in terms of severe I/R injury in the context of modern invasive management of STEMI, is still not fully understood.

Cardiac magnetic resonance (CMR) imaging is the current noninvasive gold standard technique for the in vivo evaluation of I/R injury.^14^^,^^15^ Several patterns of I/R have been described.^16^ Patients with a hemorrhagic phenotype (intramyocardial hemorrhage [IMH]) are considered to have the most severe form of I/R injury.^16^ IMH can be accurately detected by CMR T2∗ mapping based on the paramagnetic characteristics of hemoglobin degradation products.^15^ Over the last years, IMH has become of major clinical interest due to the strong prognostic value following STEMI.^15^, ^16^, ^17^ Considering these strong prognostic implications of IMH, it may also serve as a potential therapeutic target to improve outcomes after STEMI.

The aim of our study was to investigate the relationship of circulating inflammatory biomarkers with I/R injury, defined by the presence of IMH according to CMR T2∗ mapping, and clinical outcomes in STEMI patients undergoing primary PCI.

## Methods

### Study design, clinical assessments, and endpoint definitions

We analyzed 456 STEMI patients enrolled in the MARINA-STEMI (Magnetic Resonance Imaging in Acute ST-Elevation Myocardial Infarction; NCT04113356) study^18^ between 2017 and 2023. The inclusion criteria were defined as follows: first STEMI according to the European Society of Cardiology/American College of Cardiology committee criteria,^19^ revascularization by primary PCI within 24 hours after symptom onset, an estimated glomerular filtration rate >30 mL/min/1.73 m^2^, and Killip class <3 at the time of CMR. Exclusion criteria were age <18 years, history of a previous myocardial infarction or coronary intervention, fever (temperature >38 °C) or experience of an acute infection with fever in the last 14 days before study inclusion, chronic inflammatory diseases, and any contraindication to CMR (pacemaker, claustrophobia, cerebral aneurysm clip, orbital foreign body, and known contrast agent allergy to gadolinium).

Peripheral venous blood samples were obtained via peripheral venepuncture 48 ± 12 hours post-PCI. All samples were analyzed immediately after blood collection at the Central Laboratory of the Medical University of Innsbruck. Measurements of hs-CRP, IL-6, high-sensitivity cardiac troponin T (hs-cTnT), and N-terminal pro-B-type natriuretic peptide (NT-proBNP) levels were analyzed by using the Cobas 8000 Modular Analyzer (Roche Diagnostics). Sysmex XE-5000 was applied for WBCc analysis.

The presence of I/R injury was defined by the detection of IMH as determined by CMR T2∗ mapping. The primary objective of this study was to evaluate any association between the inflammatory response and the presence of IMH. Clinical endpoint was the occurrence of major adverse cardiac events (MACE) defined as composite of all-cause death, myocardial reinfarction, and new congestive heart failure, defined as cardiac decompensation with a need of intravenous diuretic therapy with or without hospitalization. In patients suffering from more than one event during the follow-up period, only the first endpoint was used for the composite endpoint. Clinical follow-up data were conducted via telephone interview using a standardized questionnaire. All interviews were completed by trained personnel blinded to CMR, laboratory, and angiographic findings. Prior study inclusion, all participants gave their written informed consent. The study received approval by the local ethics committee of the Medical University of Innsbruck and was conducted in accordance with the Declaration of Helsinki.

### Cardiac magnetic resonance imaging

CMR examinations were performed on a 1.5-T scanner (MAGNETOM Avanto^fit^, Siemens Healthineers) 4 (Q1-Q3: 2-5) days after PCI. For image acquisition and postprocessing, the standardized imaging protocol of our research group has been used.^20^

Briefly, short-axis cine images acquired by electrocardiogram-triggered trueFISP bright-blood sequences were used to evaluate left ventricular (LV) volumes and function. For postprocessing, standard software was applied (Circle Cardiovascular Imaging Inc).

For infarct characterization, electrocardiogram-triggered, phase-sensitive inversion recovery sequences were used to obtain late gadolinium enhancement images 15 to 20 minutes after application of a 0.2 mmol/kg bolus of contrast agent (Gadovist, Bayer, Leverkusen). Infarct size was quantified on a PACS workstation (IMPAX, Agfa HealthCare), by defining “hyperenhancement” as +5 SDs above the signal intensity of remote LV myocardium.^21^ Infarct size was expressed as percentage of LV myocardial mass (LVMM). Microvascular obstruction (MVO) was defined as persisting area of “hypoenhancement” within the infarcted, hyperenhanced area. IMH was determined by T2∗ mapping and was defined as region of hypointense core within the infarcted area with reduced T2∗ signal intensities below 20 ms.^15^ All postprocessing analyses were performed by experienced observers, blinded to both clinical and angiographic data.

### Statistical analysis

SPSS Statistics, 29.0 (IBM, Armonk) as well as MedCalc Version 20.115 (Ostend, Belgium) were used for statistical analyses. Distribution of data was tested using the Shapiro-Wilk test. Continuous variables are presented as mean ± SD for normally distributed data, or as median with 25th-75th percentiles (Q1-Q3) for non-normally distributed data. Categorical variables are expressed as absolute numbers with corresponding percentages. Differences in continuous variables between 2 patient groups were evaluated by Mann–Whitney *U* test, differences in categorical variables by the chi-square test. Univariable and multivariable logistic regression analyses were used to disclose significant and independent predictors of IMH. Inflammatory markers and clinical variables showing significant association (*P* < 0.10) with IMH in univariable analysis were further included into the multivariable model. In order to ensure statistical robustness given our sample size and number of events, we have conducted 4 different models: 1) inflammatory biomarkers at 48 hours; 2) inflammatory biomarkers as well as hs-cTnT and NT-proBNP at 48 hours; 3) clinical parameters, and 4) CMR parameters. To enable a better comparison of the ORs, ORs with 95% CIs are shown per 1 SD increase for continuous variables. Cox regression analysis was performed to evaluate the relation of hs-CRP and IMH with MACE presented as the HR with 95% CI. MACE-free survival was estimated and illustrated by the Kaplan-Meier method and differences were assessed by the log-rank test. A 2-tailed *P* value of <0.05 was considered as statistically significant for all statistical tests.

## Results

### Baseline patient characteristics

A total of 456 STEMI patients (18% female) with a median age of 58 (Q1-Q3: 53-67) years were included in the present study. Baseline demographics and their relationship with IMH are presented in Table 1. All patients were revascularized by primary PCI with a median ischemia time of 185 (Q1-Q3: 113-331) minutes. IMH was observed in 150 (33%) patients. There was no association between time to CMR and presence of IMH (*P* = 0.79). Patients presenting with IMH had more frequently a preinterventional TIMI flow grade 0 (81% vs 55%, *P* < 0.001) and less frequently a postinterventional TIMI flow grade 3 (83% vs 91%, *P* = 0.026). Median (Q1-Q3) hs-cTnT levels at 48 hours (3,784 [2,541-5,760] ng/L vs 1,815 [853-3,167] ng/L, *P* < 0.001) and NT-proBNP values at 48 hours (1,708 [978-3,288] ng/L vs 1,030 [538-1,856] ng/L, *P* < 0.001) were both significantly increased in patients with IMH.

**Table 1:** Patient Characteristics

	Total Population (N = 456)	IMH (n = 150, 33%)	No IMH (n = 306, 67%)	*P* Value
Age, y	58 (53-67)	58 (53-66)	58 (53-68)	0.82
Female	83 (18.2)	28 (18.7)	55 (18.0)	0.86
Body mass index, kg/m^2^	26.1 (24.3-28.6)	26.0 (24.3-28.6)	26.2 (24.3-28.5)	0.78
Current smoker	240 (52.6)	80 (53.3)	160 (52.3)	0.83
Diabetes mellitus	39 (8.6)	16 (10.7)	23 (7.5)	0.26
Hypertension	202 (44.3)	70 (46.7)	132 (43.1)	0.48
Hyperlipidemia	237 (52.0)	77 (51.3)	160 (52.3)	0.85
Total ischemia time, min	185 (113-331)	205 (119-338)	174 (111-330)	0.29
Anterior infarct localization	218 (47.8)	73 (48.7)	145 (47.4)	0.80
Preinterventional TIMI flow grade 0	289 (63.4)	121 (80.7)	168 (54.9)	**<0.001**
Postinterventional TIMI flow grade 3	402 (88.2)	125 (83.3)	277 (90.5)	**0.026**
hs-CRP 48 h, mg/L	26.4 (12.8-48.2)	39.1 (23.2-73.2)	20.2 (11.3-37.5)	**<0.001**
WBCc 48 h, g/L	8.8 (7.5-10.6)	9.6 (8.0-11.5)	8.5 (7.2-10.5)	**<0.001**
IL-6 48 h, ng/L	14.3 (8.4-24.9)	19.8 (11.4-39.8)	13.2 (8.0-21.5)	**<0.001**
hs-cTnT 48 h, ng/L	2,451 (1,228-4,062)	3,784 (2,541-5,760)	1,815 (853-3,167)	**<0.001**
NT-proBNP 48 h, ng/L	1,183 (638-2,247)	1,708 (978-3,288)	1,030 (538-1,856)	**<0.001**
CMR parameters				
LVEDV, mL	166 (138-194)	182 (149-206)	163 (132-187)	**<0.001**
LVESV, mL	85 (66-108)	99 (79-124)	78 (61-100)	**<0.001**
LVEF, %	48 (41-55)	44 (38-50)	51 (44-55)	**<0.001**
Infarct size, % of LVMM	16 (8-26)	25 (17-33)	12 (5-19)	**<0.001**
MVO, yes/no	264 (57.9)	147 (100)	116 (37.9)	**<0.001**
MVO, % of LVMM	0.4 (0.0-2.3)	2.5 (1.3-4.8)	0.0 (0.0-0.6)	**<0.001**

Values are median (Q1-Q3), compared with Mann-Whitney *U* test, or n (%), compared with chi-squared test. *P* < 0.05 is defined as significant and in **bold**.

CMR = cardiac magnetic resonance; hs-CRP = high-sensitivity C-reactive protein; hs-cTnT = high-sensitivity cardiac troponin T; IL = interleukin; IMH = intramyocardial hemorrhage; LVEDV = left ventricular end-diastolic volume; LVEF = left ventricular ejection fraction; LVESV = left ventricular end-systolic volume; LVMM = left ventricular myocardial mass; MVO = microvascular obstruction; NT-proBNP = N-terminal pro-B-type natriuretic peptide; WBCc = white blood cell count.

Regarding CMR parameters, patients with IMH had a significantly larger median (Q1-Q3) infarct size (25% [17%-33%] vs 12% [5%-19%] of LVMM, *P* < 0.001), larger extent of MVO (2.5% [1.3%-4.8%] vs 0% [0.0%-0.6%] of LVMM, *P* < 0.001) as well as a higher frequency of MVO (100% vs 38%, *P* < 0.001).

### Association of inflammation and intramyocardial hemorrhage

**Figure 1:**
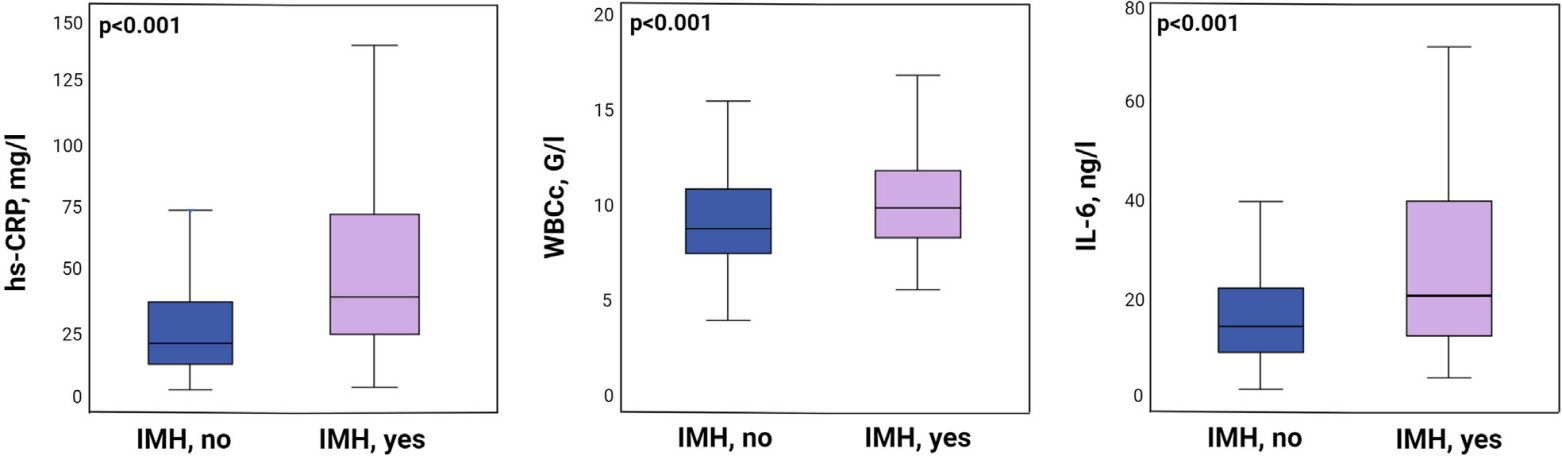
Inflammatory Parameters in Relation to Intramyocardial Hemorrhage Inflammatory parameters including hs-CRP, WBCc, and IL-6 at 48 hours after PCI in relation to IMH. hs-CRP = high-sensitivity C-reactive protein; IL = interleukin; IMH = intramyocardial hemorrhage; PCI = percutaneous coronary intervention; WBCc = white blood cell count. ∗Data are presented as median (Q1-Q3) and compared with Mann-Whitney *U* test.

Median (Q1-Q3) levels of hs-CRP, WBCc, and IL-6 at 48 hours were 26.4 (12.8-48.2) mg/L, 8.8 (7.5-10.6) g/L, and 14.3 (8.4-24.9) ng/L, respectively. Presence of IMH was significantly associated with higher 48 hours median Q1-Q3 levels of hs-CRP (39.1 [23.2-73.2] mg/L vs 20.2 [11.3-37.5] mg/L, *P* < 0.001), WBCc (9.6 [8.0-11.5] g/L vs 8.5 [7.2-10.5] g/L, *P* < 0.001), and IL-6 (19.8 [11.4-39.8] ng/L vs 13.2 [8.0-21.5] ng/L, *P* < 0.001) (Figure 1).

In multivariable regression analysis, only hs-CRP was significantly associated with presence of IMH (OR: 2.32; 95% CI: 1.47-3.65; *P* < 0.001) (Table 2).

**Table 2:** Logistic Regression Analysis for the Prediction of IMH

	Univariable Analysis	*P* Value	Multivariable Analysis	*P* Value
	OR (95% CI)		OR (95% CI)	
Model A, inflammatory markers 48 h				
hs-CRP 48 h, mg/L	2.89 (1.96-4.26)	**<0.001**	2.32 (1.47-3.65)	**<0.001**
WBCc 48 h, g/L	1.32 (1.04-1.67)	**0.02**	1.07 (0.87-1.32)	0.50
IL-6 48 h, ng/L	1.86 (1.38-2.51)	**<0.001**	1.29 (0.92-1.80)	0.14
Model B, biomarkers 48 h				
hs-CRP 48 h, mg/L	2.89 (1.96-4.26)	**<0.001**	1.80 (1.10-2.96)	**0.020**
WBCc 48 h, g/L	1.32 (1.04-1.67)	**0.021**	1.06 (0.85-1.31)	0.62
IL-6 48 h, ng/L	1.86 (1.38-2.51)	**<0.001**	1.06 (0.77-1.44)	0.74
hs-cTnT 48 h, ng/L	2.58 (2.00-3.33)	**<0.001**	2.26 (1.72-2.96)	**<0.001**
NT-proBNP 48 h, ng/L	1.52 (1.24-1.86)	**<0.001**	1.01 (0.79-1.30)	0.93
Model C, clinical parameters				
hs-CRP 48 h, mg/L	2.89 (1.96-4.26)	**<0.001**	1.95 (1.30-2.93)	**0.001**
hs-cTnT 48 h, ng/L	2.58 (2.01-3.33)	**<0.001**	2.03 (1.56-2.65)	**<0.001**
Preinterventional TIMI flow grade 0	0.29 (0.18-0.46)	**<0.001**	0.46 (0.27-0.77)	**0.003**
Postinterventional TIMI flow grade 3	0.52 (0.30-0.93)	**0.027**	0.84 (0.45-1.60)	0.60
Model D, CMR parameters				
hs-CRP 48 h, mg/L	2.89 (1.96-4.26)	**<0.001**	1.60 (1.02-2.50)	**0.041**
LVEF, %	0.53 (0.43-0.66)	**<0.001**	0.98 (0.75-1.28)	0.88
Infarct size, % of LVMM	3.38 (2.59-4.41)	**<0.001**	3.03 (2.24-4.10)	**<0.001**

*P* < 0.05 is defined as significant and in **bold**.

Abbreviations as in Table 1.

Besides inflammatory markers, hs-cTnT and NT-proBNP at 48 hours showed a significant association with the presence of IMH (3,784 [Q1-Q3: 2,541-5,760] ng/L vs 1,815 [Q1-Q3: 853-3,167] ng/L, *P* < 0.001 and 1,708 [Q1-Q3: 978-3,288] ng/L vs 1,030 [Q1-Q3: 538-1,856] ng/L, *P* < 0.001, respectively). In a second multivariable model, including inflammatory markers at 48 hours as well as hs-cTnT and NT-proBNP at 48 hours (Table 2), hs-CRP remained significantly associated with IMH (OR: 1.80; 95% CI: 1.10-2.96; *P* = 0.020). In a clinical model (Table 2), including hs-cTnT, preinterventional and postinterventional TIMI flow, hs-CRP still remained independently associated with IMH (OR: 1.95; 95% CI: 1.30-2.93; *P* = 0.001). Also in the CMR model (Table 2), hs-CRP remained significantly related with the presence of IMH (OR: 1.60; 95% CI: 1.02-2.50; *P* = 0.041) after adjustment for left ventricular ejection fraction and infarct size.

### Clinical outcomes

**Figure 2:**
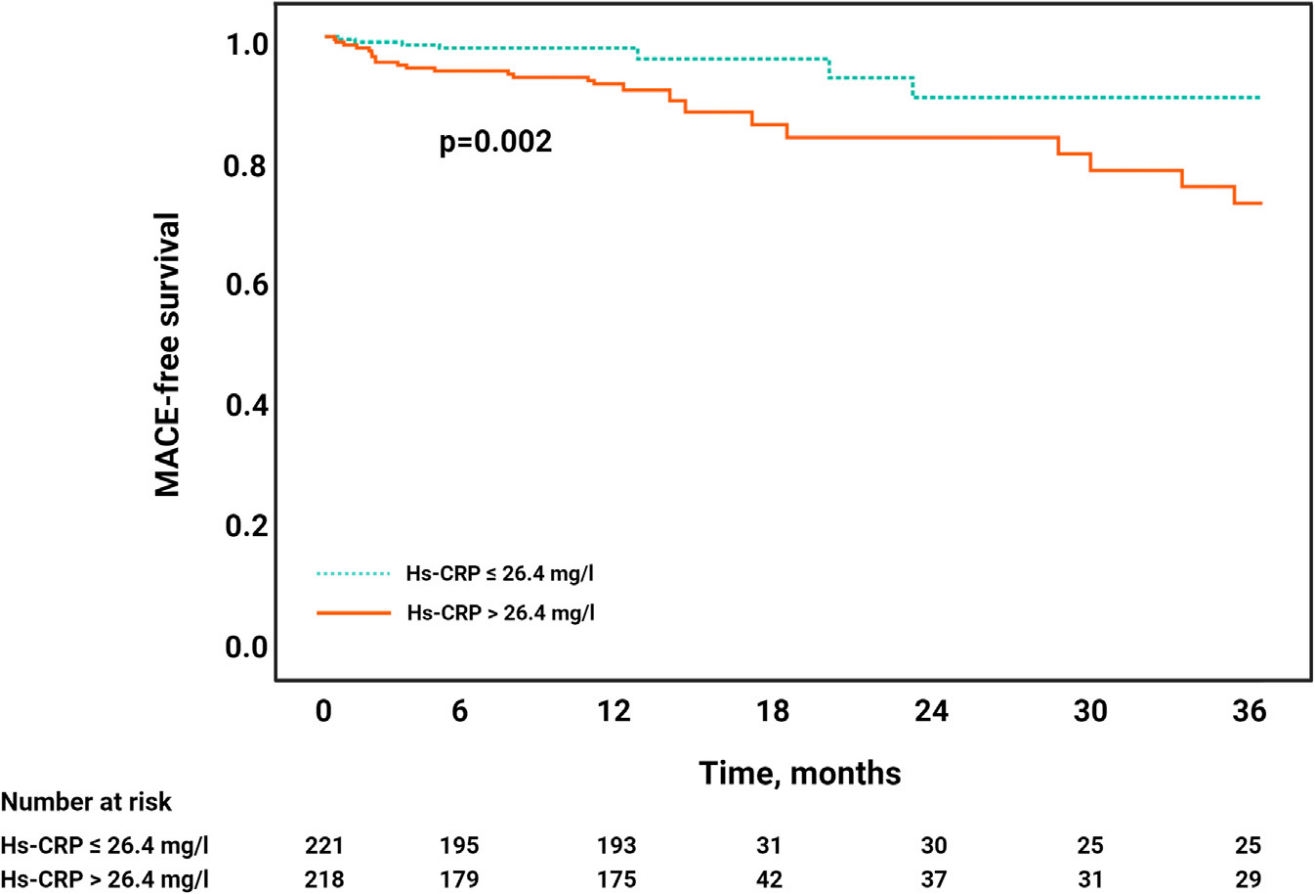
Kaplan-Meier Curves Displaying Major Adverse Cardiac Events-Free Survival Kaplan-Meier curves displaying MACE-free survival in relation to hs-CRP at 48 hours. MACE = major adverse cardiac events; other abbreviation as in Figure 1.

Follow-up data were available in 439 (96%) patients. During a median follow-up period of 12 (Q1-Q3: 12-13) months, 36 (8%) patients experienced a MACE (11 deaths; 10 myocardial reinfarctions; 15 new congestive heart failures). In Cox regression analysis, hs-CRP levels above the median (>26.4 mg/L) significantly predicted the occurrence of MACE (HR: 3.31; 95% CI: 1.51-7.29; *P* = 0.003). As illustrated by the Kaplan-Meier curves, patients with hs-CRP concentrations above the median (>26.4 mg/L) at 48 hours post-STEMI showed a significantly lower MACE-free survival (*P* = 0.002) (Figure 2).

## Discussion

In the present CMR study, we observed a significant association between circulating inflammatory biomarkers and I/R injury, defined by the presence of IMH as assessed by CMR T2∗ mapping. In particular, hs-CRP levels at 48 hours after PCI emerged as independent inflammatory biomarker associated with IMH. Furthermore, patients with hs-CRP above the median (>26.4 mg/L) showed a significantly lower MACE-free survival. Taken together, our study shows that hs-CRP is independently associated with pathophysiological processes of failed reperfusion of myocardial tissue and subsequently with an increased MACE rate. These data further support the role of hs-CRP in early risk stratification after PCI for STEMI and suggest that CRP could also be a therapeutic target to minimize I/R damage. This is a hypothesis that warrants further research.

I/R injury is a complex phenomenon involving various factors, all of which contribute to the final myocardial damage.^22^ The mechanisms involved include oxidative stress, inflammation, and calcium overload which can further exacerbate myocardial injury.^22^ IMH is a central pathological consequence of severe I/R injury with irreversible capillary destruction and subsequent extravasation of erythrocytes into the myocardium.^17^ CMR imaging is the most accurate method for the “in vivo” characterization of postinfarction myocardial injury. By incorporating newer techniques, CMR offers enhanced infarct characterization, including the visualization of necrotic core featuring hemorrhage.^23^ In particular, T2∗ mapping is the preferred technique for the in vivo quantification of IMH.^24^ Of note, detection of IMH is of considerable importance due to the close relation with adverse cardiac remodeling and subsequent worse outcome after STEMI.^15^ Moreover, a recent study demonstrated that patients with a hemorrhagic phenotype of I/R injury (IMH) had significantly more future adverse events compared to patients without IMH.^16^ In the current study, IMH was present in 33% of the population which is in line with previous findings.^15^^,^^17^ The exact pathophysiological mechanisms linking IMH and worse prognosis remain incompletely understood. Of note, Liu et al^25^ described a more rapid infarct expansion and significantly higher rates of infarct transmurality in patients with IMH compared to patients without IMH in the first 24 hours after reperfusion. A key role might be an exaggerated inflammatory response due to toxic hemoglobin degradation products which further promotes persisting inflammation and fibrosis.^26^

The infarcted myocardium inflammatory response is a prerequisite for scar formation and depends on timely suppression and narrowing down of inflammation.^27^^,^^28^ Prolonged inflammation leads to more severe damage of the myocardial tissue, myocardial dysfunction, and subsequent adverse outcome.^29^ Increased endothelial permeability and subsequent recruitment of inflammatory markers may also play a role in exacerbating I/R injury.^22^^,^^30^ CRP represents a key acute-phase protein in the setting of myocardial infarction and is mainly produced by the liver after stimulation of several cytokines, including IL-6.^31^^,^^32^ This is in line with our study, showing significantly elevated CRP levels in the acute setting after STEMI. Increased CRP levels have been associated with larger infarct size, LV dysfunction, and subsequent adverse clinical outcomes following myocardial infarction.^7^^,^^33^, ^34^, ^35^ Furthermore, experimental evidence demonstrated that CRP was deposited together with complement within the infarct area indicating a potential role in augmenting infarct size through complement activation.^36^ In addition, inhibition of exogenous added human CRP reduced infarct size in rats suggesting CRP as a potential target to reduce the extent of myocardial damage.^36^ A small clinical study provided promising results by lowering CRP with CRP apheresis and thereby showing a reduction in infarct size, however, this investigation was a pilot study and warrants further validation.^37^ In our study, CRP levels were significantly higher in patients with presence of IMH. This association of CRP and IMH in the present study remained significant after adjustment for other inflammatory biomarkers and also for multiple clinical parameters. Of note, even after adjustment for CMR determined left ventricular ejection fraction and infarct size, hs-CRP remained independently related with the presence of IMH. Although some previous studies evaluated the association between inflammatory biomarkers and I/R injury, it is important to note that they had very small sample size and most often used conventional T2 imaging instead of T2∗ mapping for IMH assessment.^38^, ^39^, ^40^ Our study therefore confirms and expands these findings by demonstrating, in a much larger sample size, that inflammatory markers (especially CRP) are associated with severe I/R injury post-STEMI. Importantly, we were also able to show that this association remained significant after adjustment for multiple other factors. Our findings further underscore the hypothesis that targeting CRP in the setting of acute myocardial infarction might minimize myocardial damage. In particular, the question arises whether these high-risk patients could benefit from early and specific intervention aimed at lowering CRP after PCI. Whether CRP is only an associate or a causal driver in the complex processes of I/R injury remains an important research question for future studies.

IL-6 acts as a pro-inflammatory cytokine and represents a main inducer of CRP.^41^ IL-6 concentrations are significantly higher in STEMI patients and have also been linked to microvascular dysfunction and adverse outcome.^6^^,^^42^^,^^43^ We can confirm those prior results by showing significant higher values of IL-6 in patients with presence of IMH. In the present analysis, however, the association of IL-6 with IMH remained not significant after adjustment for CRP. Nevertheless, clinical trials showed promising data by inhibiting IL-6 with a monoclonal antibody and demonstrating an increased myocardial salvage index as well as a reduced extent of MVO in STEMI patients receiving IL-6 inhibition.^44^ Whether IL-6 inhibition also has an influence on the occurrence of IMH is currently unclear and should be investigated in future studies.

Acute myocardial infarction triggers the recruitment of pro-inflammatory leukocytes which are essential for the reparative process leading to replacement of the infarcted area by collagen.^45^ Although inflammation is essential for proper healing, a dysregulation in this well-defined cascade leads to widespread tissue injury.^46^ An exaggerated inflammatory response might result in increased microvascular permeability and leukocyte accumulation.^45^ Experimental observations illustrated a correlation between the amount of leukocytes and the extent of infarct area.^47^^,^^48^ The aforementioned observations have also been supported by clinical studies where, for example, Palmerini et al^49^ demonstrated that elevated WBCc on admission are significantly related to CMR determined infarct size 30 days after PCI. With our study, we can expand these results by revealing significantly higher WBCc levels at 48 hours in the presence of IMH. However, in multivariable analysis, only CRP emerged as independent inflammatory parameter for the prediction of IMH.

We could also illustrate that patients with increased CRP levels had significantly higher MACE rates. This is in line with a large meta-analysis comprising 18,715 individuals, where Liu et al^10^ demonstrated that elevated CRP levels are a significant predictor of MACE events in patients with acute myocardial infarction undergoing PCI. Our findings therefore further emphasize the role for CRP in terms of optimized risk stratification after primary PCI for STEMI. Those with high risk as indicated by CRP could not only benefit from novel adjunctive therapies on top of standard of care but also from prolonged monitoring and closer follow-up after hospital discharge.

### Study Limitations

The following limitations must be considered: First, We cannot prove causality by study design. Second, biomarkers were assessed at 48 hours after PCI, thus, the study could not evaluate the optimal time point for biomarker assessment or the potential differences in their release kinetics. Further research is needed to address this important gap of knowledge. Third, our findings are not generalizable to hemodynamically unstable STEMI patients as only stable STEMI patients with Killip class I and II at the time of CMR were included.

## Conclusions

In acute STEMI patients revascularized by primary PCI, hs-CRP was significantly and independently associated with I/R injury as defined by the presence of IMH (Central Illustration). Our findings therefore provide novel pathophysiological insights into the association of inflammation and failed myocardial tissue reperfusion in post-STEMI.

Perspectives **COMPETENCY IN MEDICAL KNOWLEDGE:** Identification of patients with distinct pathophysiological alterations is important for the implementation of effective therapy strategies. Thus, biomarker-based approaches are essential to identify individuals exhibiting excessive inflammatory responses. With the present study, we can provide significant and independent association between increased CRP levels at 48 hours and presence of IMH after STEMI.

**TRANSLATIONAL OUTLOOK:** Experimental data suggested that inhibiting C-reactive protein in acute myocardial infarction reduces myocardial damage. Further investigation is warranted as to whether patients with high C-reactive protein levels could benefit from targeted interventions.

**Central Illustration:**
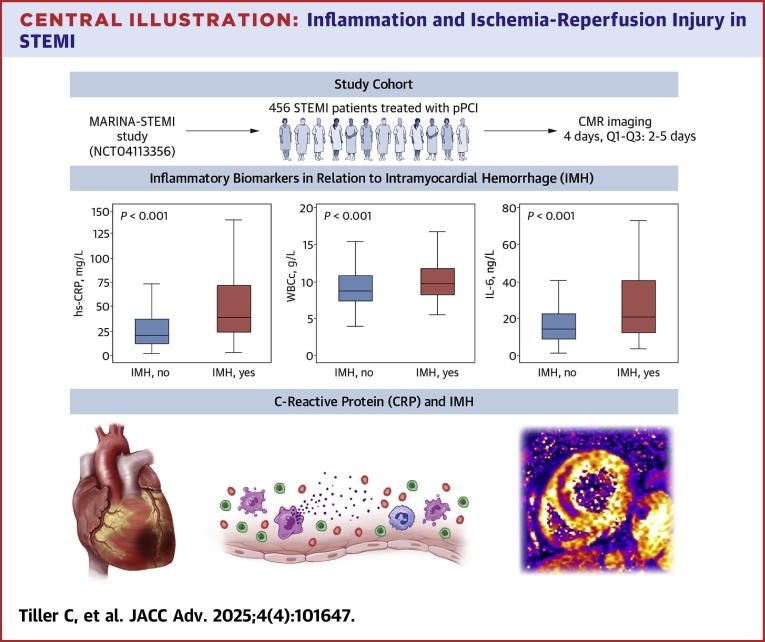
Inflammation and Ischemia-Reperfusion Injury in STEMI Central Illustration summarizing the association of several inflammatory biomarkers with intramyocardial hemorrhage. CMR = cardiac magnetic rresonance; pPCI = primary percutaneous coronary intervention; other abbreviations as in Figure 1.

## Funding support and author disclosures

This study was supported by grants from the “10.13039/501100015797Austrian Society of Cardiology,” the “Gesellschaft zur Förderung der Herz-Kreislauf-Forschung,” and the “10.13039/501100010591Tiroler Wissenschaftsfonds.” The authors have reported that they have no relationships relevant to the contents of this paper to disclose.
